# Lights, camera, active! appreciation of active learning predicts positive attitudes towards lecture capture

**DOI:** 10.1007/s10734-020-00674-4

**Published:** 2021-01-18

**Authors:** Emily Nordmann, Anne Clark, Elliott Spaeth, Jill R. D. MacKay

**Affiliations:** 1 School of Psychology, University of Glasgow, G12 8QB Glasgow, Scotland; 2 Learning Enhancement and Academic Development, University of Glasgow, G12 8LB Glasgow, Scotland; 3 Royal Dick Veterinary School, University of Edinburgh, EH25 9RG Edinburgh, Scotland

**Keywords:** Lecture capture, Lecture recordings, Pedagogical approach, Active learning, Student centered, Teacher centered

## Abstract

Much has been written about instructor attitudes towards lecture capture, particularly concerning political issues such as opt-out policies and the use of recordings by management. Additionally, the pedagogical concerns of lecturers have been extensively described and focus on the belief that recording lectures will impact on attendance and will reduce interactivity and active learning activities in lectures. However, little work has looked at the relationship between attitudes towards lecture capture and broader conceptions of learning and teaching. In this pre-registered study, we administered the Conceptions of Learning and Teaching scale and a novel lecture capture attitude scale to 159 higher education teachers. We found that appreciation of active learning predicted more positive attitudes towards lecture recordings as an educational support tool, whilst higher teacher-centred scores predicted greater concern about the negative educational impact of recordings. The effects observed were small; however, they are strong evidence against the view that it is instructors who value participatory and active learning that are opposed to lecture capture. Exploratory analyses also suggested that those who did not view recordings as an essential educational resource record fewer of their lectures, highlighting the real-world impact that attitudes can have, and further strengthening the need for staff to be provided with evidence-based guidance upon which to base their teaching practice. Data, analysis code, and the pre-registration are available athttps://osf.io/uzs3t/.

## Introduction

The process of recording some aspect of a teaching activity, often called ‘lecture capture’, has existed in higher education for several decades, being a mainstay of distance learning provision such as in the UK’s Open University, and Australian universities’ support for English as a Second Language (ESL) and outreach students (Evans, 2008; Scutter et al., 2010; The Open University, n.d.). Students have largely positive views about lecture capture, but staff have a number of concerns surrounding how it affects the teaching and learning environment (Kwiatkowski & Demirbilek, 2016; Nordmann et al., 2019). This conflict between staff and student perceptions can make lecture capture a disruptive technology, and challenge its introduction to higher education institutions (MacKay, 2019).

There have been concerns that lecturers with a more interactive teaching style may become more rigid and less collaborative when recording lectures (Bond & Grussendorf, 2013; MacKay, 2019) although Gosper et al. (2010) found that 39% of academics claimed that they had not altered their lecturing style substantially as a result of being recorded and only 8% claimed to have adopted a more didactic lecture style, suggesting that while the introduction of lecture capture is a source of concern and anxiety for educators, it is not necessarily a force for change. As for the impact on students, there is mixed evidence about how learning is impacted by lecture capture. Lecturers commonly consider the personal interaction in lectures a fundamental aspect of the pedagogical design (Kwiatkowski & Demirbilek, 2016). One concern is that some studies have found that students self-report that they may attend lectures less frequently if recordings are provided (Gorissen et al., 2012; Leadbeater et al., 2013; Owston et al., 2011), although observational studies and meta-analyses have found no consistent effect on attendance (Nordmann & McGeorge, 2018; O’Callaghan et al., 2017). Student attainment is thought to be improved by lecture captures through students having greater control of the materials, allowing for deeper engagement, particularly with challenging topics (Dey et al., 2009; Toppin, 2011). The relationship with achievement is more complex; some studies have found that lecture capture has a limited effect on those whose grades were already good, whilst students who have been struggling, or have barriers to engagement make much higher use of recordings (Leadbeater et al., 2013; Owston et al., 2011) whilst others have found an interaction between GPA and attendance whereby use of lecture recordings only improves performance for weaker students if used as a supplement, rather than a substitute, to live lectures (Nordmann et al., 2019). Other perceived benefits are both emotional and practical. Students experience less anxiety about their coursework when having lecture captures available (Nordmann & McGeorge, 2018; O’Callaghan et al., 2017). In addition, lecture capture has proven useful in inclusive education allowing students with specialized learning needs and students with outside commitments (e.g., working students, students with families) as well as second language learners to review material at their own pace (Gosper et al., 2010; Nordmann et al., 2019). Lecture capture appears to have positive benefits, particularly for those students who may experience barriers to entering higher education, but it is less clear how and why educators choose to engage with the technology, and how their attitudes may affect uptake.

How an educator conceives of teaching and learning in turn affects their teaching (Bolhuis & Voeten, 2004; Windschitl, 2002), and so attitudes to teaching may have an important impact on lecture recording usage. Attitudes to teaching and learning can be broadly characterised as ‘teacher-centred’, where the teacher is viewed as the expert responsible for the conveyance of information to the students, and ‘student-centred’, where the student is viewed as the primary actor who directs their learning often in discussion and co-construction with the educator (Elen et al., 2007; Packer & Goicoechea, 2000). Teacher-centred versus student-centred models of teaching and learning mainly differ in their epistemological and ontological assumptions, with student-centred models coming from socio-constructivist principles, and teacher-centred learning coming from more positivist backgrounds (Bransford et al., 2005; Yuen & Hau, 2006). Positivism, which focuses on content rather than student learning, is not a popular outlook within educational theories, and yet is a highly prevalent epistemology, particularly within STEMM practitioners (Matthews, 2004) despite the fact that STEMM subjects have also led the way in student-centred teaching such as the flipped classroom (Draper et al., 2018).

The relationship between these pedagogical approaches and attitudes towards lecture capture is unclear. As noted above, descriptive studies on staff attitudes often report that educators believe recording lectures will reduce interaction (Bond & Grussendorf, 2013; Gosper et al., 2010; MacKay, 2019; O’Callaghan et al., 2017), and so one may hypothesise that it is those who favour a student-centred, active approach to learning that would be most strongly against lecture capture. The competing prediction is that the more student-centred approach to teaching and learning may be favoured by those who recognise barriers to higher education provision and wish to support student learning through the provision of additional resources that promote flexibility. In this light, lecture capture may be one of many resources, and the lecture one of many learning opportunities that can support a more diverse group of students including those with learning disabilities (Nightingale et al., 2019).

One of the most widely used instruments for measuring conceptions of learning and teaching is the Approaches to Teaching Inventory (ATI; Trigwell & Prosser, 1996). Trigwell and Prosser (1996) based the statements for this inventory on an earlier qualitative study (Trigwell et al., 1994) of first-year university lecturers in which they were able to confirm a link between teachers’ conceptions of teaching and student learning and their approach to teaching, with more teacher-centred conceptions related to more traditional ‘data transmission’ or didactic teaching methods. The ATI has subsequently been used in many small-scale studies across several disciplines with similarly trending results; however, as it is course-specific, it is not always clear how comparable the results are across disciplines (Trigwell & Prosser, 1996). In addition, much of the ATI was developed with a limited trialling, resulting in concerns with its validity (Meyer & Eley, 2006). An alternative instrument is Conceptions of Teaching and Learning (COLT) to measure three underlying factors, ‘teacher centredness’, ‘appreciation of active learning’, and ‘orientation to professional practice’ (Jacobs et al., 2012). The COLT instrument was developed and successfully used to measure different conceptions to teaching among the staff at two medical colleges. It was able to differentiate the conceptions of teaching and learning between professors at one university who were new to student-focused teaching and those at another sister university who had never taught in a different way. Those new to student-focused teaching maintained teacher-centred conceptions and still saw themselves as purveyors of knowledge. COLT has been further used to characterise educators as ‘transmitters’ with highly traditional lecturing styles, to ‘conceptual change agents’ who embrace new technologies (Jacobs et al., 2014).

Many of the concerns around lecture capture reveal a surprising contradiction in lecturers’ perception of the purpose of lectures, where they are both a one-time live performance and opportunity for students to experience an expert in the field, but also something that should not be canonised and repeated by students (MacKay, 2019). How these attitudes relate to more fundamental conceptions of teaching may help to explain the concerns staff have with lecture recording, and guide policy makers and academic developers to better support and integrate lecture capture usage in higher education. In this study, we made use of COLT to assess lecturers’ fundamental attitudes to teaching and learning and employed a new scale to explore attitudes to lecture recording, to characterise the relationships between the use of the tool and conceptualisations of teaching and learning within lecturers working in higher education. This new scale was constructed to measure attitudes towards lecture capture that concern pedagogical issues, rather than more political aspects such as policy or the use of recordings for performance management. Aside from the issue of attendance or canonicity, much of the literature on staff attitudes to lecture capture has focused on these political aspects (e.g., Dommett et al., 2019) and whilst these issues are important, there is a need to consider a broader pedagogical view of lecture capture to help educators understand the decisions they face and make. We hypothesised that those with higher student-centred scores would express more positive views towards lecture capture. Although the concerns of some that recordings may make lecturers more rigid and less collaborative (Bond & Grussendorf, 2013) may suggest the opposite relationship, this hypothesis is driven by acknowledging the interaction with constructivist vs. positivist beliefs; that is, the disruptive potential of lecture capture is likely greater for those educators whose teaching places more importance on didactic lectures than those who believe learning happens through co-creation and active learning. The main hypothesis and analysis were pre-registered, although given the novel scale, our study should still be considered exploratory.

## Method

The study design, hypotheses, and analyses were pre-registered and can be viewed at https://osf.io/uzs3t/, including details of any deviations.

### Participants

Ethical approval for this study was obtained from the Board of Ethics of the College of Science and Engineering at the University of Glasgow, and recruitment was conducted via internal and external (e.g., JISC) mailing lists and social media (primarily the Twitter and LinkedIn accounts of the authors). Participants from all career stages were invited to participate regardless of whether they recorded any lectures; the only criteria for participation was that they delivered at least one HE lecture per year. In total, 199 participants took part (mean age = 42.83, SD = 9.49, range 21–63, missing = 7), see Table 1 for demographic information. Of these participants, 159 fully completed all three sections. Our sample was within the pre-registered range of 136 to 182 participants, based on an effect size of *f*^*2*^ 0.06–0.10.

**Table 1:** Participant demographic information

Region	Percent	Gender	Percent	Field of study	Percent	Teach in native language	Percent
UK	78.39	Female	50.75	Arts	14.57	Yes	89.45
Europe	5.03	Male	41.21	Medical, veterinary and life sciences	16.58	No	10.55
Oceania	13.07	Non-binary	2.01	Science and engineering	39.70		
Asia	1.51	Missing	6.03	Social sciences	26.53		
N. America	2.01			Academic development	2.51		

**Fig. 1:**
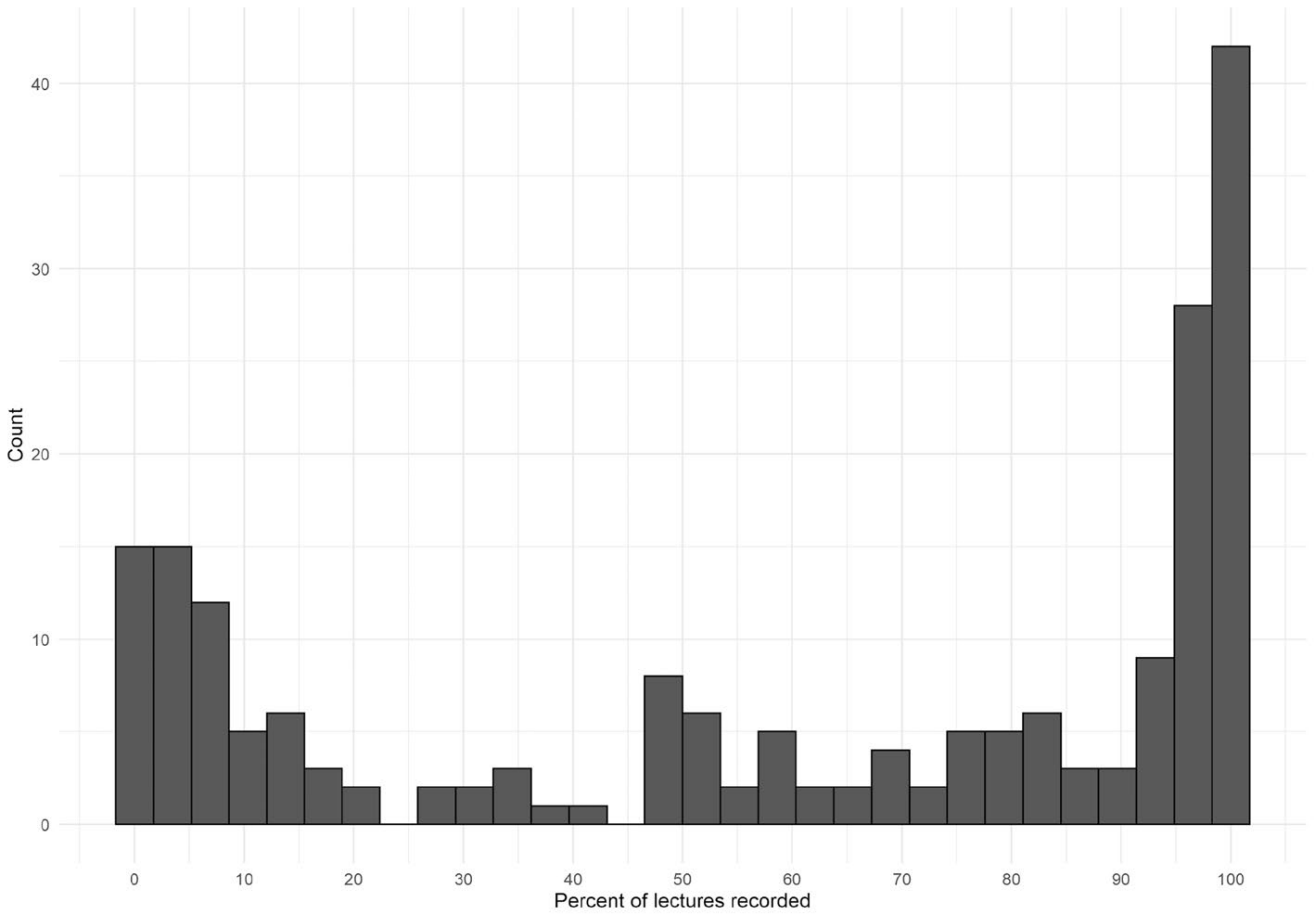
Distribution of percent of recorded lectures per lecturer

On average, participants had 13.61 years of teaching experience and delivered 35 lectures per year (see Table 2). The average percent of lectures recorded was 60.21%; however, it is important to note that this statistic masks a polarised bimodal distribution with approximately 29% of participants recording less than 20% of their lectures and approximately 40% recording 90% or more (see Fig. 1).

**Table 2:** Participant teaching information

	Mean	SD	Median	Min	Max	Skew	Kurtosis
Teaching experience (years)	13.61	8.44	12	1	37	0.61	− 0.42
Lectures per year	34.54	23.73	30	1	100	1.16	0.96
Percent of lectures recorded	60.21	39.27	75	0	100	− 0.42	− 1.50

### Materials and procedure

A questionnaire consisting of 3 blocks (demographics, conceptions of teaching and learning, beliefs about lecture recordings) was hosted on the University of Glasgow Experimentum platform (DeBruine, n.d.).

#### Demographic questions

The first block of demographic questions asked participants to provide their age, gender, country, field of study, teaching experience, number of lectures delivered per year, percent of lectures recorded, and whether they taught in their native language. The presentation of the second and third blocks was randomised, and all statements within these blocks were randomised to avoid order effects. A full list of the items can be found in the online supplementary information.

#### Conceptions of learning and teaching

The second block measured conceptions of learning and teaching (COLT; Jacobs et al., 2012). The 18 COLT statements appeared in random order, and participants were asked to indicate their level of agreement using a five-point Likert scale (1—completely disagree to 5—completely agree). Eight of the 18 COLT statements were reverse coded. The COLT scale contained three subscales. The first, Teacher Centredness, had 8 items (e.g., ‘*Students learn best when the learning process is guided by an expert who has an overview of the field of interest*’); the second (5 items), Appreciation of Active Learning, had statements such as ‘*Small group learning motivates students to study*’; and the last subscale (5 items), ‘Orientation to Professional Practice’, had items such as ‘*It is a good learning experience when students demonstrate they can apply their knowledge to real-life situations*’. The original COLT questions were specifically designed to be administered to medical educators, and therefore, minor adaptations were made to four of the questions to make them applicable to a general audience, for example ‘*Being introduced to the day-to-day practice of their future profession motivates students to learn*’ was modified to ‘*Being introduced to the day-to-day practice of their future profession and/or to practical applications of their studies helps students to learn*’. Permission to use and modify the COLT statements was obtained from the authors (Jacobs et al., 2012).

#### Beliefs about lecture capture

The third block was a novel scale to measure beliefs about lecture capture (BALC). Fifteen items were developed by the first and second authors based upon common concerns and beliefs reported in previous qualitative research, as well as adhering to the ultimate/proximate distinction described by MacKay (2019) and refined by the third and fourth authors. The items were shown in random order, and participants indicated their agreement on a five-point Likert scale with six of the 15 statements was reverse coded. The BALC items were developed to map broadly on to two sub-scales. The first (six items) were those items we considered to relate to proximate (in-class) concerns about lecture recordings (e.g., ‘*Providing lecture recordings negatively affects attendance*’); the second (nine items) to ultimate concerns (e.g., ‘*Providing lecture recordings encourages students to review lecture content they found difficult to understand*’), however, as specified in the pre-registration; and the final sub-scale structure was determined through principal components analysis (PCA). The items in the original sub-scales with their analysis labels are available in the online supplementary information.

### Data analysis

The structure and reliability of the BALC were investigated and confirmed first through PCA and then by exploratory factor analysis, with Cronbach’s alpha calculated for the final sub-scales. To test the main hypotheses, regression models were constructed with the mean BALC sub-scales as the outcome variable and the mean COLT sub-scale scores as predictors. The assumptions of the parametric regression models were tested and corrected for, and these models were also confirmed by equivalent ordinal regression modelling with Wald tests for significance. Finally, a number of exploratory analyses were conducted to investigate if there was any evidence for relationships with demographic variables. 

## Results

All analyses were conducted using R and RStudio (Version 1.2.5033, RStudio Team, 2019) using the packages tidyverse (Wickham et al., 2019), psych (Revelle, 2019), pwr (Champely, 2020), broom (Robinson & Hayes, 2020), lsr (Navarro, 2015), knitr (Xie, 2015), kableExtra (Zhu, 2019), ggraph (Pedersen, 2020), car (Fox & Weisberg, 2019), ggforce (Pedersen, 2019), ordinal (Christensen, 2019), afex (Singmann et al., 2020), and emmeans (Lenth, 2020). All deviations from the pre-registration are described in full in the online supplementary information.

### Scale construction and validation

#### Descriptive statistics

**Fig. 2:**
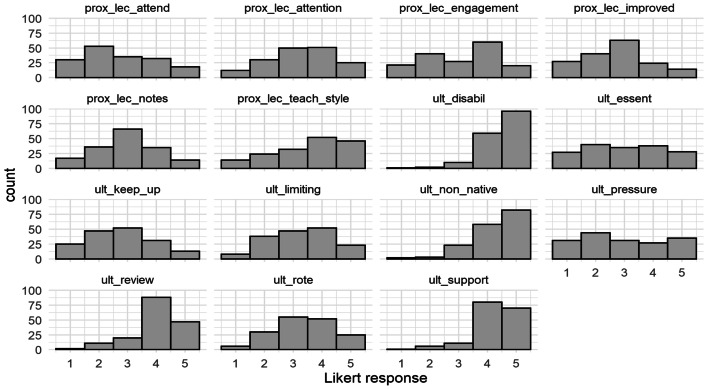
Distributions for the BALC scale items

Descriptive statistics and the distribution of responses for each item can be seen in Table 3 and Fig. 2. In total, 168 participants fully completed the BALC section. Of note, responses to the items suggesting that recordings were useful for students with disabilities, non-native speakers, as a supplementary resource, and as a revision tool were negatively skewed with most respondents expressing agreement with these statements.

**Table 3:** Descriptive statistics for the BALC scale items

	Mean	SD	Median	Min	Max	Skew	Kurtosis
prox_lec_attend	2.73	1.26	3	1	5	0.30	− 1.00
prox_lec_attention	3.28	1.14	3	1	5	− 0.24	− 0.73
prox_lec_engagement	3.11	1.25	3	1	5	− 0.22	− 1.14
prox_lec_improved	2.75	1.14	3	1	5	0.18	− 0.60
prox_lec_notes	2.96	1.08	3	1	5	0.00	− 0.53
prox_lec_teach_style	3.55	1.26	4	1	5	− 0.53	− 0.79
ult_disabil	4.47	0.72	5	1	5	− 1.54	3.23
ult_essent	3.00	1.34	3	1	5	0.01	− 1.21
ult_keep_up	2.76	1.15	3	1	5	0.19	− 0.76
ult_limiting	3.26	1.10	3	1	5	− 0.13	− 0.85
ult_non_native	4.28	0.85	4	1	5	− 1.19	1.43
ult_pressure	2.95	1.42	3	1	5	0.14	− 1.31
ult_review	3.99	0.88	4	1	5	− 0.99	1.01
ult_rote	3.36	1.05	3	1	5	− 0.16	− 0.67
ult_support	4.26	0.78	4	1	5	− 1.23	2.05

#### Principal components analysis

All 15 items correlated at least 0.3 with most of the other items, the Kaiser-Meyer-Olkin measure of sampling adequacy was 0.90, and Barlett’s test of sphericity was significant (*χ*^2^(105) = 1134.43, *p* < 0.001) suggesting that the data were suitable for PCA. PCA was conducted using the ‘principal’ function from the ‘psych’ package (Revelle, 2019).

An initial model using the number of items as factors (as per Field et al., 2012) was constructed. Based upon the scree plot and eigenvalues, a two-component model was selected. A second model was then constructed with two components using varimax rotation (see Table 4). This model accounted for 54% of the total variance; however, there were two problematic items, ‘ult_essent’ and ‘prox_lec_improved’ which did not load cleanly on either scale and so were removed. Following a qualitative review of the remaining items, we also decided to remove ‘prox_lec_teach_style’ and ‘ult_pressure’. Whilst they loaded cleanly on component 2, they are incongruous as the only items that specifically related to the teacher, rather than the impact on learning. The final model accounted for 58.19% of the variance, and this component structure was replicated using exploratory factor analysis (see online supplementary information). Whilst the variance explained is lower than anticipated and suggests that further scale development would be beneficial, it is in line with Peterson (2000) who found that the average PCA variance in peer-reviewed papers was 56.6% and approaches Hair et al’s (2010) suggestion for a minimum of 60% variance explained for social science measures.

**Table 4:** Two-component PCA loadings: (a) all items and (b) reduced items

	Component 1a	Component 2a	Component 1b	Component 2b
prox_lec_attend	0.30	− 0.70	0.30	− 0.70
prox_lec_attention	0.36	− 0.67	0.35	− 0.72
prox_lec_engagement	0.55	− 0.29	0.54	− 0.31
prox_lec_improved	0.49	− 0.50		
prox_lec_notes	0.66	− 0.36	0.66	− 0.39
prox_lec_teach_style	0.33	− 0.66		
ult_disabil	0.81	− 0.14	0.82	− 0.14
ult_essent	0.56	− 0.43		
ult_keep_up	0.60	− 0.29	0.59	− 0.27
ult_limiting	0.29	− 0.64	0.28	− 0.71
ult_non_native	0.83	− 0.10	0.84	− 0.13
ult_pressure	0.10	− 0.72		
ult_review	0.78	− 0.17	0.79	− 0.15
ult_rote	− 0.02	− 0.61	− 0.03	− 0.70
ult_support	0.78	− 0.15	0.78	− 0.16
Prop. variance %	43.59	10.46	46.10	12.09
Cumulative variance %	43.59	54.06	46.10	58.19
Cronbach's α	0.87	0.83	0.86	0.74

The components with their items are in Table 5. The original proximate/ultimate categories were not reflected in the final item groupings; however, on review, the PCA sub-scales suggested a distinction between individual and higher-level concerns. The items of the first component all refer to study skills and the use of lecture recordings for individual students and so we refer to this scale as the educational support sub-scale, with higher scores reflecting the belief that lecture recordings are a useful educational support tool for a variety of reasons. The second sub-scale items are all focused on negative impacts of lecture recordings including those that go beyond individual students and have impact at a course level such as attendance and so we refer to this scale as the recording impact scale.

**Table 5:** Final sub-scale items with component groupings

Item	Scale
I would not/do not mind if students fail to attend some of the live lectures as long as they engage with the recordings throughout the semester	Educational support
Providing lecture recordings encourages students to take better, paraphrased notes rather than writing down what the lecturer says verbatim	Educational support
Providing lecture recordings is beneficial to students with, e.g., learning disabilities and physical and mental health problems	Educational support
Providing lecture recordings is beneficial to students with English as a second language, etc.	Educational support
Providing lecture recordings encourages students to review lecture content they found difficult to understand	Educational support
Supplemental use of lecture recordings in addition to lecture attendance can help support learning	Educational support
Providing lecture recordings encourages students to keep up with the lecture content throughout the semester, rather than cramming near exam-time	Educational support
Providing lecture recordings negatively impacts attendance	Recording impact
Providing lecture recordings negatively impacts how much students pay attention in the live lecture	Recording impact
Providing lecture recordings discourages students from looking beyond the lecture for additional information (e.g., reading)	Recording impact
Providing lecture recordings encourages students to rote learn the lecture content	Recording impact

### Predictors of beliefs about lecture recordings

Descriptive statistics, including Cronbach’s alpha, for each of the sub-scales are shown in Table 6. For both the BALC and the COLT, 168 participants completed each section; however, only 159 participants completed both scales and the remaining analyses are therefore conducted upon these complete cases.

**Table 6:** Descriptive statistics for BALC and COLT sub-scales

	Mean	SD	Median	Min	Max	Skew	Kurtosis	Alpha
record_support	3.71	0.73	3.71	1.00	5.00	− 0.85	1.04	0.86
record_impact	2.85	0.85	2.75	1.00	4.50	− 0.03	− 0.80	0.74
active_learning	4.10	0.43	4.20	2.80	5.00	− 0.30	0.03	0.49
professional_practice	4.28	0.49	4.20	2.20	5.00	− 0.62	0.88	0.64
teacher_centred	2.89	0.56	2.88	1.25	4.25	− 0.12	− 0.13	0.76

**Fig. 3:**
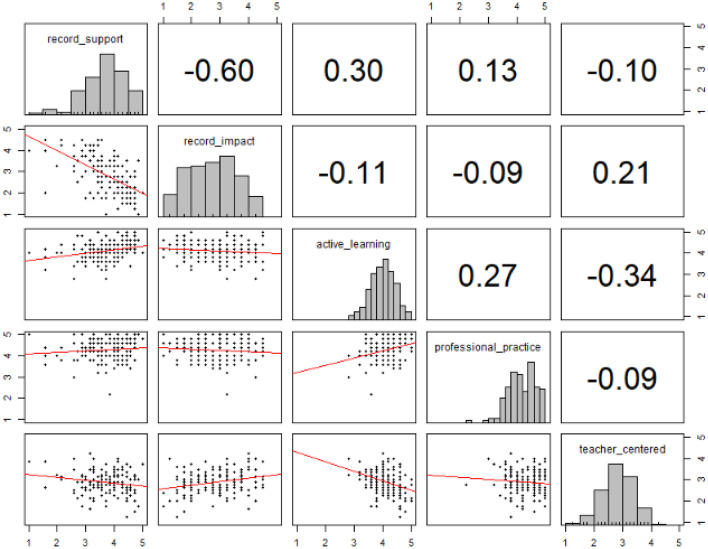
Scatterplots and histograms with Spearman correlation coefficients

Orientation to professional practice and appreciation of active learning scores were generally high and negatively skewed whilst teacher-centred scores were lower and normally distributed (see Fig. 3). Scores on the educational support scale were higher and more normally distributed than those on the negative impact scale, which were lower and approached a uniform distribution. Of note, Cronbach’s alpha for the COLT sub-scales, particularly the active learning and orientation to professional practice, are lower than the suggested lower-bound of 0.70 although they are in line with the original alphas reported in Jacobs et al. (2012).

#### Correlations between the sub-scales

Spearman’s correlations were conducted between the sub-scale scores of the BALC and COLT (see Table 7 and Fig. 3). There was a negative correlation between the educational support scores and impact of recordings scores, and a positive relationship between the educational support scores and appreciation of active learning scores. There was no relationship between negative impact scores and the COLT scales, although the relationship with the teacher-centred scores was marginal after multiple comparison correction (*p* = 0.052). For the internal COLT correlations, active learning scores were negatively correlated with teacher-centred scores, and positively correlated with orientation to applied practice.

**Table 7:** Spearman’s correlations for the BALC and COLT sub-scales

	record_support	record_impact	active_learning	professional_practice
record_impact	− 0.60**			
active_learning	0.30*	− 0.11		
professional_practice	0.13	− 0.09	0.27*	
teacher_centred	− 0.10	0.21	− 0.34**	− 0.09

*p < 0.01; **p < 0.001

#### Regression analyses

To aid interpretability and comparison with the literature, parametric regression models were constructed and validated with ordinal regression which provided an identical pattern of results (see online supplementary information). Two regression models were constructed with educational support and negative impact scores as the outcome variables, and the three COLT sub-scales as predictors. Regression assumptions were checked using a combination of statistical tests (Shapiro-Wilk, non-constant variance, variance inflation) and visualisations (see online supplementary information for detailed results). The negative impact model passed all tests; however, the educational support model failed the assumption of normally distributed residuals due to substantial negative skew. As per Tabachnick, Fidell and Ullman (2007), a log10 transformation was applied to the outcome variable, educational support scores. This transformed model passed all assumption tests.

*Attitudes towards lecture recordings as an educational support tool.* The overall regression model for the educational support sub-scale was significant with a small effect size (*F*(3, 155) = 5.888, *p* < 0.001, adjusted *R*^2^ = 0.09, *f*^2^ = 0.093, see Table 8). Scores on the active learning sub-scale were a positive predictor of educational support scores; scores on the orientation to professional practice and teacher-centred sub-scales were not significant.

**Table 8:** Regression table for the educational support model (log transformed)

	Estimate	Std error	*t*	*p*	Lower CI	Upper CI
Intercept	0.709	0.149	4.764	0.000	0.415	1.003
active_learning	− 0.085	0.027	− 3.196	0.002	− 0.138	− 0.032
professional_practice	− 0.015	0.022	− 0.689	0.492	− 0.059	0.029
teacher_centred	0.016	0.020	0.794	0.428	− 0.023	0.054

*Beliefs about the negative impact of lecture recordings.* The overall regression model for the negative impact sub-scale was significant with a small effect size (*F*(3, 155) = 3.842, *p* = 0.01, adjusted *R*^2^ = 0.05, *f*^2^ = 0.054, see Table 9). Scores on the teacher-centred sub-scale were a positive predictor of negative impact scores, whilst scores on the orientation to professional practice and active learning sub-scales were not significant predictors.

**Table 9:** Regression table for the negative impact model

	Estimate	Std error	*t*	*p*	Lower CI	Upper CI
intercept	2.491	0.956	2.607	0.010	0.604	4.379
active_learning	− 0.039	0.171	− 0.230	0.818	− 0.377	0.298
professional_practice	− 0.125	0.142	− 0.880	0.380	− 0.407	0.156
teacher_centred	0.364	0.126	2.883	0.004	0.115	0.614

### Exploratory analyses

In addition to the main analyses that were pre-registered, a number of exploratory analyses were conducted to investigate any potential relationships between the BALC sub-scales and teaching-related demographic variables. For brevity, only significant results are described in full here; however, full results can be found in the online supplementary information.

**Fig. 4:**
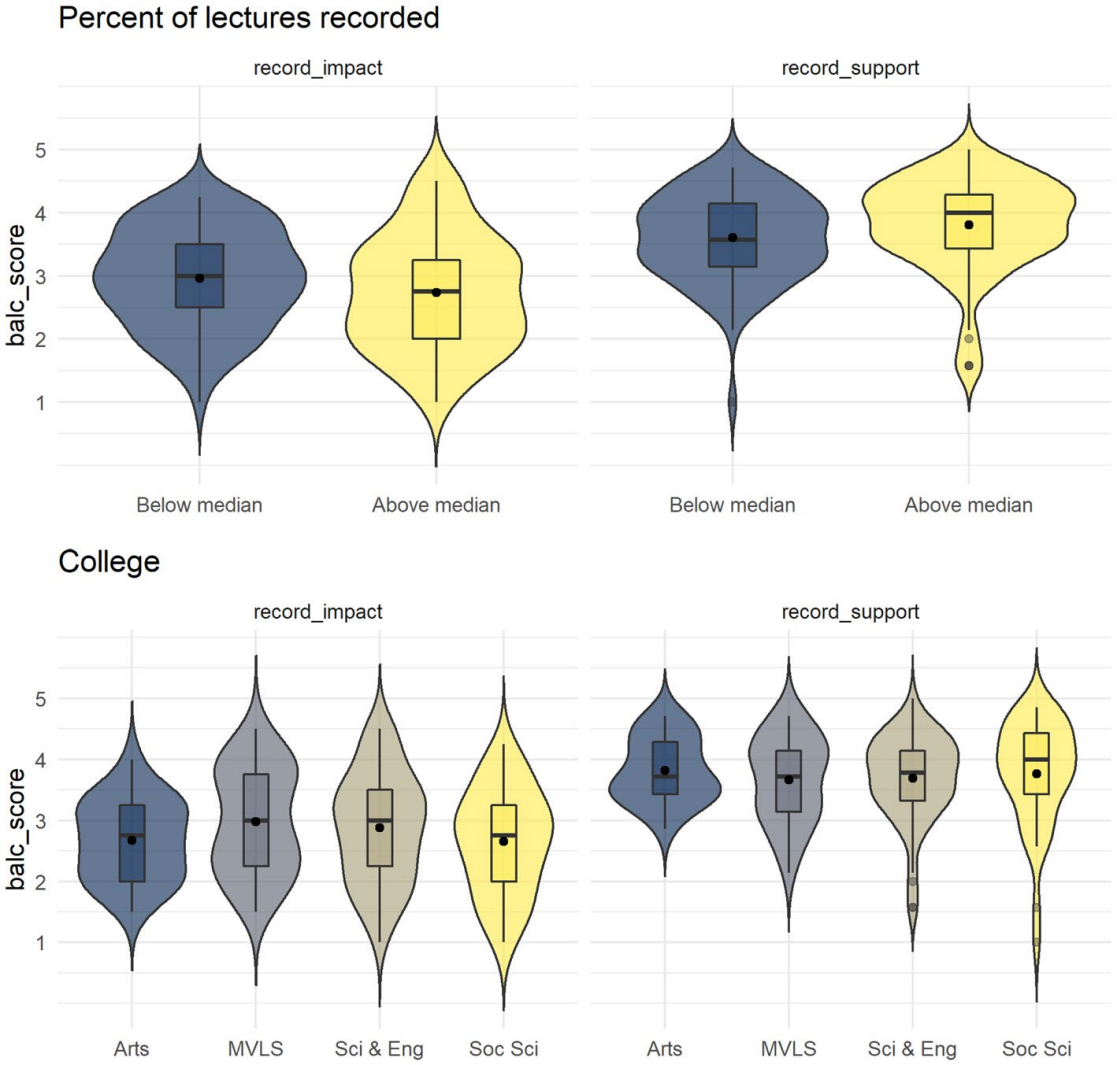
Violin-boxplots for BALC sub-scale scores by **a** lecture recording status and **b** subject college

No relationship was found between any of the BALC or COLT sub-scales and teaching experience or number of lectures given per year. Given the bimodal distribution of responses to the question about the percent of lectures recorded, this variable was transformed into a categorical variable based on a median split. No difference on either BALC sub-scale was found between participants who indicated they recorded more or less than the median percent of recorded lectures (see Fig. 4a). Participants’ responses to their field of study were recoded into four colleges based on the structure of the University of Glasgow; Arts, Medical, Veterinary and Life Sciences, Science and Engineering, and Social Science. There were no differences on either of the BALC sub-scales between colleges (see Fig. 4b).

**Fig. 5:**
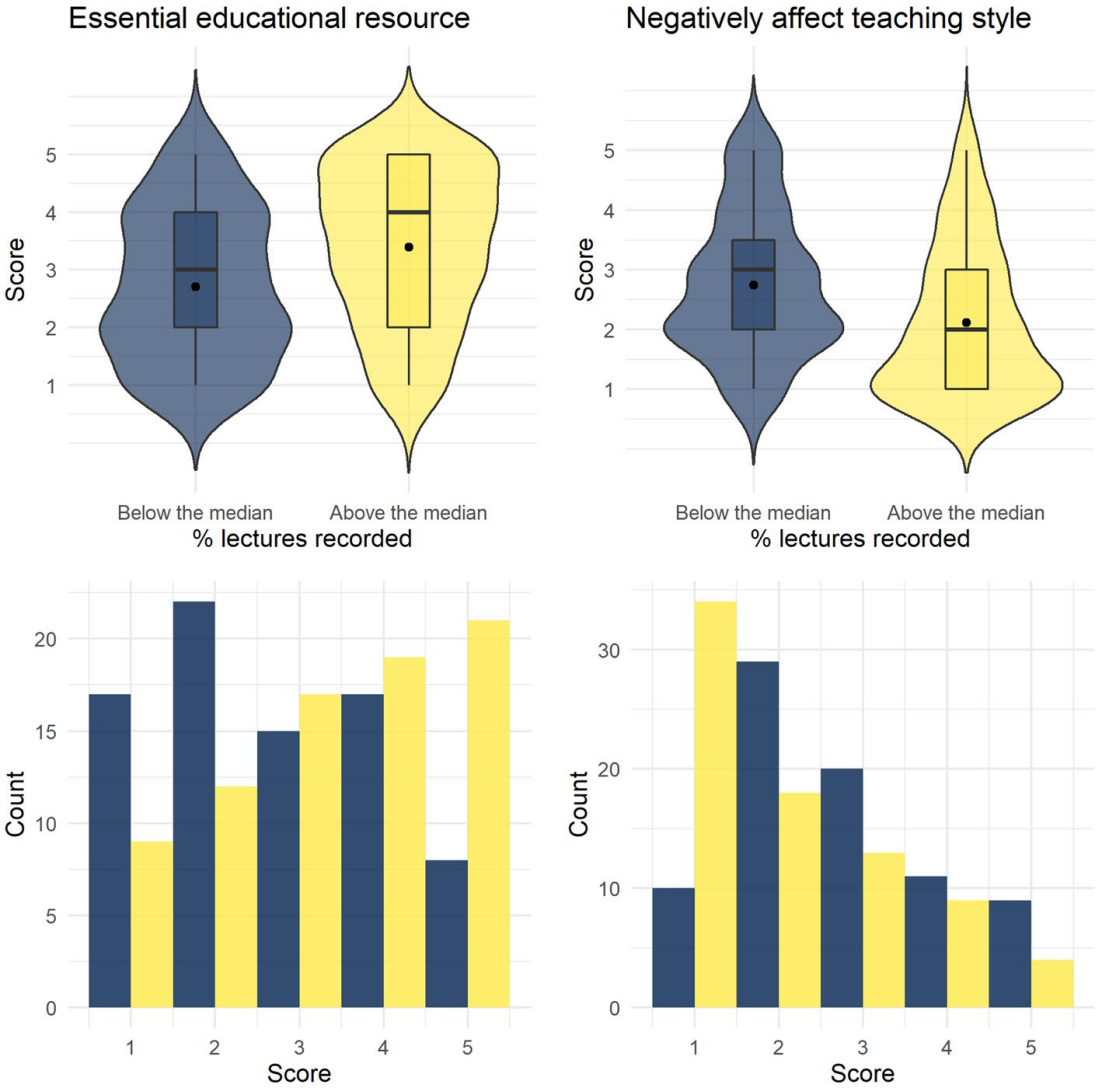
Violin-boxplots and histograms for the exploratory regression outcomes. Higher scores equate to higher agreement with the scale items

Finally, exploratory analyses were conducted on the items that were removed from the BALC scale during the PCA validation. Scores on each individual item were used as the outcome variable with the COLT sub-scales and demographic variables (college, teaching experience, lectures per year, percent lectures recorded (categorical)) as predictors. For the items ‘Providing lecture recordings would/has improved the way I deliver lectures’ and ‘I feel/would feel pressured to provide lecture recordings’ neither the overall models nor any of the individual predictors were significant. For ‘Providing lecture recordings would/does negatively affect my teaching style’ the outcome was log-transformed for positive skew. The overall model was not significant although given the *p* value is still worth highlighting (*F*(9, 147) = 1.9, *p* = 0.056, adjusted *R*^2^ = 0.049, *f*^2^ = 0.103). The categorical lecture capture recording variable was significant as an individual predictor (*β* = 0.143, *p* < 0.001) with those who recorded below the median percent of lectures being more likely to agree that recordings would affect teaching style. Finally, for ‘I consider lecture recordings an essential educational resource and I expect my students to use them if available’ the overall model was significant (*F*(9, 147) = 2.791, *p* = 0.005, adjusted *R*^2^ = 0.094, *f*^2^ = 0.103) with the categorical recording variable as the sole significant predictor (*β* = 0.772, *p* < 0.001) with those who recorded above the median being more likely to agree that recordings were an essential educational resource (see Fig. 5). All exploratory regressions were validated with ordinal regression.

## Discussion

In this study, we used the COLT and a novel scale, BALC to explore higher education teachers’ attitudes to teaching, learning, and lecture recording. Our key findings were that educators with a teacher-centred approach to learning were more likely to hold negative opinions on the impact of lecture recording on teaching. By contrast, those with positive beliefs about active learning were more likely to hold positive beliefs about the educational support lecture recordings can provide and the exploratory analyses suggested that these attitudes are not divided along discipline lines. How can these findings be used to best support teaching and learning in future?

A common concern surrounding the recording of lectures is that it will reduce participation in classrooms (MacKay, 2019). With student participation the core focus of active learning, this concern may initially appear at odds with our finding that it is educators who favour active learning who hold more positive views on the impact of recording on teaching, and that, conversely, teacher-centred educators, who prioritise transmission of content over active participation, are more likely to be concerned about negative impacts of lecture capture. If transmission of content from the teacher is the key ingredient of their lectures, what lies behind the assumption that recording them would negatively impact student learning?

Student engagement in education is a complex meta-construct, consolidating aspects of learning success, student attainment, and student satisfaction (Fredricks et al., 2004). Engagement has behavioural and psychological components and can indicate the students’ assimilation into academic culture (Kahu, 2013). However, it is how student participation and engagement are perceived by teacher-centred educators that are particularly relevant when considering their answers to survey questions. Attendance and perceived attention are two commonly used indicators of participation and engagement, and perhaps it is those that are being considered by our teacher-centred educators; hence, the worry that students will feel there is less need to attend lectures or concentrate throughout the lecture as the recording will be available. Students are considered to spend a great deal of time in lectures in ‘off-task’ activities (Ragan et al., 2014), which educators may attribute to the existence of recordings that can be viewed later.

Engagement is more than attendance, and so reductions in attendance to lectures, the effect of which is debatable, is arguably not a true problem for student participation if the student is able to engage in other ways, i.e., through external reading, labs, tutorials, etc. Indeed, lecture capture can facilitate engagement by allowing students to learn in their own time, at their own pace, and in an environment that suits them. This may be especially important in relation to teacher-centred lectures, which can fall into unhelpful traps such as causing cognitive overload (Weidman & Baker, 2015) and failing to include student learning opportunities and engagement (Bond & Grussendorf, 2013; Kottasz, 2005).

Lecture capture thus opens up learning to a wide range of students who have previously been disadvantaged by face-to-face, transmissive lectures, by enabling them to use the lecture recording as well as, or instead of, attending the lecture. This flexibility is particularly meaningful for students who would find it difficult to attend, for example, due to caring responsibilities or health issues, as well as those who may find lectures a suboptimal learning environment, such as students with Attention Deficit Hyperactivity Disorder (ADHD) who may benefit greatly from being able to work at their own pace (MacKay, 2019). These are a handful of examples, but by considering the needs of specific individuals, we can create a flexible learning environment that is more supportive for everyone (Burgstahler, 2009).

Lecture capture, like many forms of Technology Enhanced Learning, has been touted as a ‘disruptive technology’ for higher education (Gosper et al., 2010; Preston et al., 2010). Disruptive technologies are those which eventually displace the established practice, possibly through offering improved experience or outcomes (Danneels, 2004). Lecture capture has often been assumed to disrupt because of its potential to replace the live lecture. To this point, MacKay (2019) identified the need for institutions to clearly specify that recordings are meant to be used to supplement live lectures and that they cannot be used to replace scheduled lectures in the event of the absence of the lecturer, e.g., during industrial action. However, the majority of evidence suggests that staff and students still highly value the interaction and that, if anything, captures will disrupt study strategies (MacKay, 2019). The complexity of university learning and teaching reflected in the lecture capture debate suggests that the most effective approach to supporting student learning may be to focus staff development conversations on pedagogy more generally, as opposed to focusing on recordings as a distinct tool. Put simply, the less that traditional didactic lecturing forms part of the teaching methods on offer, the less disruptive that lecture capture can be, in addition to the pedagogic benefits of making lectures more active. Similarly, students need to be taught general study skills that incorporate recordings. Recorded materials have been used in education for many decades (Zawacki-Richter & Naidu, 2016), and we cannot expect their use to reasonably decline, particularly in light of social changes that may be expected post COVID. Recordings are also a common method of delivering content for professional development or skills building, with resources such as Khan Academy, Skillshare, and YouTube becoming popular hosts of educational material (Clifton & Mann, 2011; Fernández et al., 2014). Nordmann et al. (2020a) provide student guidance for embedding the use of recordings into general study strategies and also provide recommendations for instructors adopting lecture recordings, including increasing the amount of active learning during traditional lectures.

An alternative lens through which to view our results is Attribution Theory (Weiner, 2010) and to what individual educators explain the success or failure of their teaching.^1^ Attribution theory proposes three causal dimensions that affect an individual’s appraisal of an outcome: locus of causality (whether the attribution has an internal or external locus of causality), stability (whether the cause is perceived to be a stable or fluctuating factor), and controllability (whether the individual has control over the factors perceived to affect the outcome). In a systematic review of the literature on attribution theory and education, Wang and Hall (2018) highlight that teachers tend to attribute student failure to internal student characteristics rather than teacher characteristics or task difficulty (e.g., Jager & Denessen, 2015; Tollefson et al., 1990) and there is a parallel to be drawn with lecture capture. Educators who attribute lecture attendance to stable, internal, controllable characteristics of students (e.g., laziness) rather fluctuating, external, uncontrollable factors (e.g., the need to work, disability) are likely to more negatively appraise the use of a technology that allows for non-attendance. Additionally, this framework can also be used to explain educator attitudes to institutional adoption of lecture capture technology. When the introduction of lecture capture is an external imposition over which educators feel they have little control, this is likely to result in more negative appraisals, regardless of the actual impact on either teacher or student.

The effects observed in the current study are undoubtedly small in nature; however, they offer strong evidence against the view that lecture capture is at odds with active learning. Additionally, the exploratory analyses suggested that there is a link between attitudes and behaviour with those with more negative attitudes towards lecture capture reporting that they recorded fewer lectures. Replication is required, causation cannot be established by the paradigm of the current study, and more work is needed particularly on the ‘how’ of how and why educators use lecture capture, but our study sheds a little more light on the ‘why’.

Despite evidence opposing the belief that lecture recording’ has a negative impact on student participation, it is educators’ own assumptions of the effects of lecture recording that are likely to inform their behaviour, in line with attribution theory. In this way, misconceptions and perceptions about lecture recording may reduce its usage, thus disadvantaging students who could have benefitted from viewing recordings. The potential impact of educator attitudes on the student experience is illustrated starkly in Díez et al., (2015) exploration of students with disabilities, in which attitudes of staff were reported as a substantial barrier to access, academic performance, and positive experiences of HE. This finding is aligned with the social model of disability (Oliver, 2013), which positions environmental features (such as attitudes, assumptions, and social conventions), rather than an individual’s impairment, as key disabling factors, and highlights the importance of uncovering staff attitudes as a first step to changing behaviour.

Once aware of staff attitudes that may contradict available evidence on lecture capture, there are two key elements to countering this potential issue. Firstly, it is vital to engage educators with the evidence surrounding lecture recording, attendance, and student participation (e.g., Nordmann et al., 2020a) so that they can better understand how their reasoning aligns with objective data. Secondly, future work is needed to explore educator concerns surrounding their own positioning as a ‘lecturer’, expanding MacKay (2019) finding that some educators see lectures ‘as a stage show’ with their performance the central focus. Unpicking emotional and identity-related elements of this concept would be a valuable contribution to the area of lecture recording and may provide insight into how educators can be supported to feel more comfortable and confident making lecture recordings available to their students.

One potential emotional factor behind resistance to lecture capture by more traditional teacher-centred educators is risk aversion or a resistance to change. Chang (2007) suggested that academics might be wary of adopting lecture capture because they could not see any benefits for themselves. Other studies (Lim & Chai, 2008; Tondeur et al., 2017; Windschitl, 2002; Windschitl & Sahl, 2002) confirm reluctance on the part of educators to fully utilise technology often due to outside pressures such as curricular requirements or institutional practices. The BALC data seem to echo the idea that lecture capture brings few benefits to the lecturer. While the participants do not believe the lecture capture negatively affects their teaching style, they also do not believe it improves their lecture style. Encouraging universities to train lecturers in how they can use lecture capture most effectively, for example, to save time in answering student questions and to actively engage with their lecture style (Joseph‐Richard et al., 2018), might enable them to see the benefits and not just the risks.

### Limitations

Firstly, and probably most importantly, academics’ conceptions of learning and teaching are likely just one of many factors that influence their relationship with lecture capture. For a variety of reasons, teachers’ conceptions of teaching and learning do not always translate into practice (Meyer & Eley, 2006; Windschitl, 2002), and more work is needed to explore how attitudes affect the behaviours exhibited in lectures. In our study, the fact that the scores on two COLT subscales, Appreciation of Active Learning and Orientation to Professional Practice, are generally positive while the Teacher Centredness items are more evenly distributed may reflect a bit of this dichotomy. While this study was pre-registered, both scales would benefit from further replication and development to confirm measurement validity across a range of practitioners.

As noted in the introduction, we purposefully chose not to focus on the political aspects of lecture recording, but it must be recognised that it has become an intensely political issue (Highton, 2018) and this may explain why our scale and models accounted for a low proportion of variance in the responses. Some of the pedagogical attitudes expressed by lecturers may be masking such political concerns, and an updated version of our scale with items that consider both political and pedagogical attitudes is likely necessary to disentangle the two issues. Practical considerations, such as the level of technical training and support provided by institutions, may also further explain lecturers’ attitudes to lecture capture and should be considered in future research. Given the impact that the decision not to record can have, particularly on disadvantaged students, it is critical to understand the predictors of attitudes towards lecture capture. It must be assumed that educators are affected by the culture around them and the messages, both overt and hidden, that management communicates and the potential for politics to override pedagogy highlights the importance of good policy in the implementation of recording technology (Nordmann & McGeorge, 2018). Additionally, the relatively poor reliability scores for the COLT sub-scales add an additional note of caution to our results and suggest that there is room for the development of a new scale given that our alphas were similar to the original scale validation study (Jacobs et al., 2012).

Finally, this work, along with the vast majority of lecture capture literature, describes a pre-pandemic time in higher education. SARS-COV-19 (COVID) has impacted both society at large and the education sector specifically, and this will likely continue in the short term (Nordmann et al., 2020b). The current higher education model has survived many potential technological disruptions, including MOOCs (Dey et al., 2009; Dowell et al., 2017). It is not clear how the societal upheaval linked with the pandemic will impact the present disruption in the higher education sector, how lecture recordings will fit into our new normal, and whether the lasting impact of the pandemic will be to completely revolutionise how we teach, or if this will simply be a short term blip in education history. What we do know is that the uptake of learning technology is likely to increase as a result of this disruption, and it is important to consider how attitudes and pedagogical beliefs shape the consequential choices educators make.

## Footnotes

1 We thank an anonymous reviewer for this suggestion.
